# Food Searching Strategy of Amoeboid Cells by Starvation Induced Run Length Extension

**DOI:** 10.1371/journal.pone.0006814

**Published:** 2009-08-28

**Authors:** Peter J. M. Van Haastert, Leonard Bosgraaf

**Affiliations:** Department of Cell Biochemistry, University of Groningen, Haren, The Netherlands; University of Arizona, United States of America

## Abstract

Food searching strategies of animals are key to their success in heterogeneous environments. The optimal search strategy may include specialized random walks such as Levy walks with heavy power-law tail distributions, or persistent walks with preferred movement in a similar direction. We have investigated the movement of the soil amoebae *Dictyostelium* searching for food. *Dictyostelium* cells move by extending pseudopodia, either in the direction of the previous pseudopod (persistent step) or in a different direction (turn). The analysis of ∼4000 pseudopodia reveals that step and turn pseudopodia are drawn from a probability distribution that is determined by cGMP/PLA2 signaling pathways. Starvation activates these pathways thereby suppressing turns and inducing steps. As a consequence, starved cells make very long nearly straight runs and disperse over ∼30-fold larger areas, without extending more or larger pseudopodia than vegetative cells. This ‘win-stay/lose-shift’ strategy for food searching is called Starvation Induced Run-length Extension. The SIRE walk explains very well the observed differences in search behavior between fed and starving organisms such as bumble-bees, flower bug, hoverfly and zooplankton.

## Introduction

The energy balance of organisms depends on the ratio of food intake and energy used for essential functions such as basal metabolism, movement, growth and reproduction. Therefore, efficient food searching strategies are key to the success of all organisms [1], [2]. The optimal search strategy may depend on the specific situation [2], [3]. In oriented searches the organism uses environmental feedback (e.g. chemotaxis) to locate and move towards target sites [4]. The emerging path shows preferential directions, even though substantial stochastic noise may be present. A systematic search is probably the optimal strategy when organisms know that food is present somewhere in a certain area, but have no directional information on target sites [5]. In most cases, however, it is not known to the organism if, where and how much food is present in the environment. In those cases random searches are probably more efficient than systematic searches [3]. In a random walk, the walker is equally likely to move in any possible direction and independent of the direction at all preceding directions [6]. Even if an organism uses a random walk for food searching, it can still improve the probability of finding target sites by changing some statistical properties of their motility behavior [3], [7]. For instance, the correlated random walk is characterized by an increased probability to continue movement in the same direction, while in a Levy walk the step length probability distribution is heavily tailed, giving rise to many short flights and few exceptionally long flights. Both correlated random walks and Levy walks improve encounter rates of target sites. In heterogeneous environments, Levy walks are more efficient than a single classical (Brownian) random walk, but less efficient than classical composite random walks (i.e. a classical random walk with large steps for relocations mixed with a classical random walk with small steps for intensive local search) [3], [7], [8]. In addition to these specific walks, the success of encounters may improve when the stochastic component of the movement is adaptive to the actual situation of the searcher. For instance, the diffusion coefficient, which incorporates speed, step size, and turning frequency, could be dependent on energy reserves, food intake, or the presence of predators [4], [9]. An efficient adaptation for food searching is the strategy ‘win-stay/lose-shift’, i.e. stay in the presence of food, and start moving when starving [10]–[12]. A detailed description of cell movement in the absence and presence of food may uncover the mechanisms of adaptive stochastic movement that cells use to improve the success of non-oriented searches.

**Figure 1:**
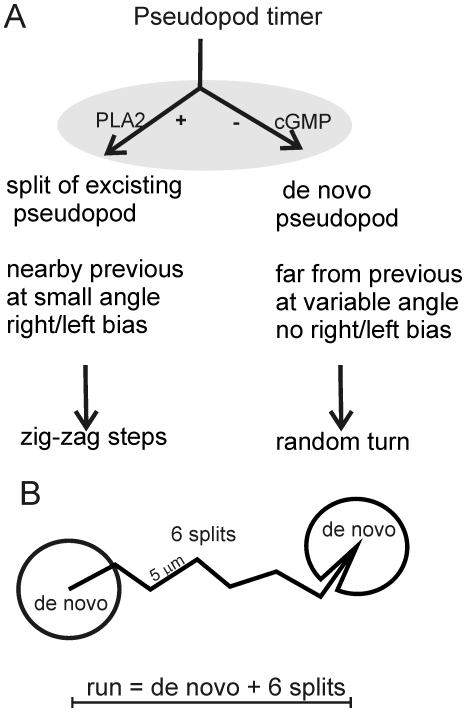
Fundamentals of *Dictyostelium* movement. A, pseudopodia. A cell extends on average one new pseudopod every ∼15 seconds. Depending on the activity of PLA2 and guanylyl cyclase, this pseudopod is formed by splitting of an existing pseudopod or formed de novo on the cell body. Splitting pseudopodia are extended at a small angle and preferentially alternating to the right and left, resulting in a relatively straight zig-zag path. In contrast, a de novo pseudopod is extended in nearly random directions. B, trajectory. Cell movement of 5 h starved cells is composed of runs that start with a turn in a random direction followed on average by ∼6 steps. The next random turn is the start of a new run. The figure is based on the ordered extension of pseudopodia in the absence of external cues [16].

We have investigated the food searching strategy of the soil amoebae *Dictyostelium*. This organism is genetically tractable to identify genes involved in food searching strategies. Eukaryotic amoeboid cells move by extending pseudopodia [13]. The size, direction and frequency of pseudopod extensions are the basis for the movement of amoeboid cells. Many eukaryote cells extend pseudopodia predominantly by splitting of an existing pseudopod and occasionally *de novo* [14]. To understand how pseudopod extension regulates cell movement, we developed a computer algorithm that identifies the size, timing and direction of extending pseudopodia [15]. We observed that splitting pseudopodia are extended preferentially alternating to the right and left giving rise to a relatively straight zig-zag run (Fig. 1). In contrast, a de novo pseudopod is extended in an approximating random direction, thereby interrupting the straight run [16]. Therefore, the movement of amoeboid *Dictyostelium* cells may be described by relatively straight runs of persistent steps mediated by pseudopod splittings and random turns by de novo pseudopodia.

This repertoire of pseudopod extensions could form the basis for food searching mechanisms of amoeboid cells. We have used the pseudopod detection algorithm to analyze in detail how *Dictyostelium* cells extend pseudopodia during starvation. We recorded long sequences of pseudopod extensions, identified the pseudopodia as split or de novo, and tested the composition of the runs to characterize the type of walk that is used by cells. The results show that the movement of cells is well described by a correlated random walk with exponential probability distribution of the run length. We obtained no statistical evidence for heavy power tail distributions or complex walks. Finally we tested the strategy that *Dictyostelium* cells use to find food. We find only one adaptation to starvation, which is the suppression of turns. This strategy leads to a ‘win-stay/lose-shift’ scenario: in the presence of food cells turn frequently and thereby do not move far, while upon starvation the suppression of turns causes large dispersion of cells in random directions.

## Results

### Dispersion of cells during starvation

**Figure 2:**
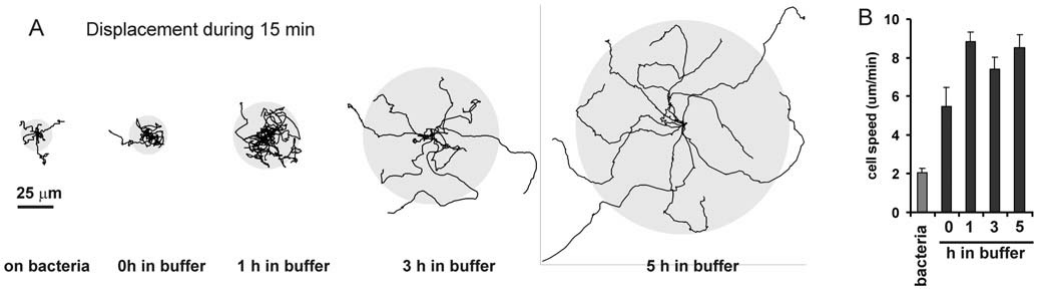
Cell dispersal during starvation. The figure shows the tracks of 10 cells during 15 min. Cells were grown on bacteria, or starved in buffer for 0 h, 1 h, 3 h or 5 h. The grey circle indicates the average root mean square dispersal. The graph shows the speed of the cells measured as instantaneous velocity of the cells' centroid at 8 s interval; data are the means +/− SEM of ∼15 cells.

Amoeboid cells such as neutrophils in the blood stream or *Dictyostelium* in the soil eat bacteria [17], [18]. With ample food supply, cell movement may not be required to engulf bacteria. Indeed, *Dictyostelium* cells on a bacteria-rich medium move at a low speed of ∼2 µm/min (Fig. 2B). However, after 1 hour of food shortage, cells can move at a relatively high speed of ∼8 µm/min. Nevertheless, cells starved for 1 hour do not disperse over a large area (∼1500 µm^2^ during 15 minutes; Fig. 2A); the cell-tracks exhibit many turns and cells repeatedly visit the same position. Cells starved for prolonged times disperse over a much larger area (∼20,000 µm^2^ after 5 h of starvation), and the cells rarely return on their path. The trajectories of 5 h starved cells show many relatively long and nearly straight runs. Strikingly, the speed of the cells is constant between 1 h and 5 h of starvation. Thus, starved cells can search for food in a larger area, not by increasing their speed, which would cost a lot of energy, but by moving longer in the same direction. We will address cells starved up to 1 h as vegetative cells.

### Random selection of split and de novo pseudopodia

The tracks of *Dictyostelium* cells are characterized by runs of steps in the same direction made by splitting pseudopodia, which are separated by turns in a new direction mediated by a de novo pseudopod. The length of the runs is therefore determined by the probability that cells extend split or *de novo* pseudopodia [16]. The extension of pseudopodia as steps or turns was recorded for 10 vegetative cells from 2 independent movies giving a total of 426 pseudopodia in 211 runs. To collect data for a similar number of runs of starved cells, we analyzed 40 starved cells from 7 independent movies yielding information on 1645 pseudopodia in 257 runs. On average, vegetative cells extend 1.8 steps and 1.6 turns per minute, giving a total pseudopod frequency of 3.4/min. Cells starved for 5 hours do not extend many more pseudopodia (3.9/min), but extend much more steps (3.4/min) relative to turns (0.5/min). The increase of the step/turn ratio in starved cells strongly influences the length of the nearly straight runs; the mean run length increased from 2.1 pseudopodia in vegetative cells (1 turn and 1.1 steps) to 7.0 in starved cells (1 turn and 6.0 steps).

**Figure 3:**
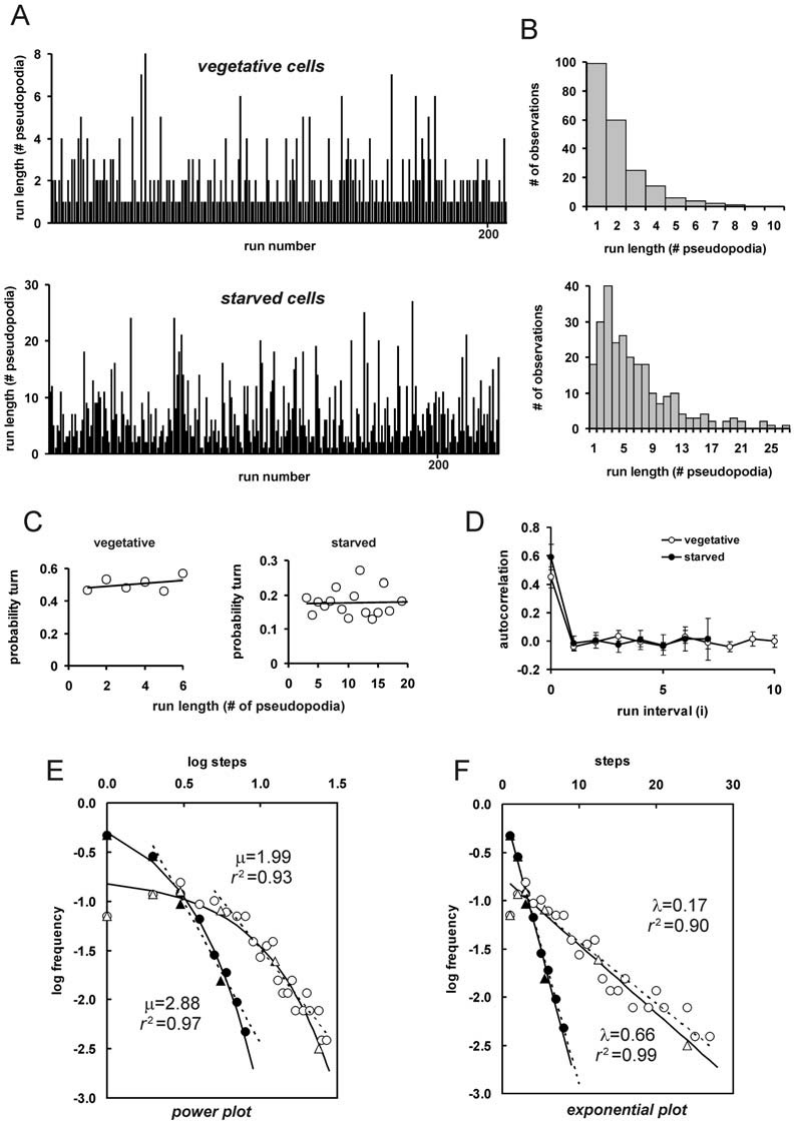
Statistical analysis of *Dictyostelium* food searching behavior. A, a run is a random turn by a *de novo* pseudopod followed by a series of persistent steps by pseudopod splitting till the next turn. The run length was determined for 10 vegetative cells and 40 starved cells during ∼15 minutes, and presented as run-length frequency distributions in (B). The mean run length is 2.1+/−1.4 and 7.0+/−5.3 for vegetative and starved cells, respectively. Panels C present the probability of a turn at increasing run length (see equation 1 in methods for calculations). Panel D presents the serial autocorrelation of run length deviation from the mean at increasing interval between runs (see equation 2 in methods for calculations); the data show the means and SEM. The constant turn frequency at increasing runs (C) and the absence of significant autocorrelation (D) suggests that turn selection is time-independent (a Bernoulli trial with random selection of turns). The data support neither a Levi walk (few exceptionally long runs resulting in heavy tails of the turn probability), nor a composite walk (clusters of short walks and long walks resulting in a positive autocorrelation of nearby runs). E, F. Power (E) and exponential plot (F) of the run-length frequency distributions. Closed and open symbols refer to vegetative and starved cells, respectively. Triangles represent log-bins of run length frequencies (1, 2, 3–4, 5–8, 9–16, 17–32 pseudopodia), while the frequencies for all sizes of run length are presented as circles. The dashed lines represent linear regression to the tails of unbinned data (run>1 and >4 for vegetative and starved cells respectively); the slope yields the power exponent μ, or the exponent λ for an exponential plot. The drawn lines are the result of a model for a Bernoulli trial with random turns and observed mean run length of 2.1 and 7.0 for vegetative and starved cells, respectively (see appendix S1 in supplemental information).

We have investigated whether the extension of a split or de novo pseudopod is random, or that it may exhibit some form of selection as in special random walks. A Levi walk has a heavy tailed distribution of long runs, implying a reduced probability to make a turn when the run becomes very long. Composite random walks are characterized by periods of short runs and periods of longer runs. The frequency of steps (s) and turns (t) was determined. The observed frequencies of the pairs ss, st, ts and tt are very close to the predicted values for a random distribution (Table 1). Furthermore, the probability to make a turn does not change with increasing run-length (Fig. 3C), which would occur in Levi walks. Finally, the serial autocorrelation analysis of the run length deviation from the mean is not significantly different from zero for any run interval (Fig. 3D), indicating no signs for clusters of short or long runs (see also methods for explanation). These data indicate that the probability of turns is time-independent, and follows a Bernoulli trial: The pseudopod is either a split or de novo, and its identity is drawn randomly from a split/de novo distribution.

**Table 1:** Frequencies of split and turn pseudopodia in vegetative and starved Dictyostelium cells.

Pseudopod	Frequencies in vegetative cells		Frequencies in starved cells	
	Observed	Calculated	Observed	Calculated
Split	0.535+/−0.057		0.838+/−0.033	
Turn	0.465+/−0.057		0.162+/−0.033	
Split-split	0.283+/−0.081	0.286+/−0.043	0.693+/−0.074	0.702+/−0.039
Split-turn	0.264+/−0.047	0.245+/−0.041	0.140+/−0.040	0.136+/−0.028
Turn-split	0.263+/−0.052	0.245+/−0.041	0.143+/−0.039	0.136+/−0.028
Turn-turn	0.190+/−0.081	0.216+/−0.038	0.024+/−0.012	0.026+/−0.008

Data are means and SD with n = 10 vegetative cells (426 pseudopodia and 410 pairs of pseudopodia) or n = 40 starved cells (1645 pseudopodia and 1598 pairs of pseudopodia).

A Bernoulli trial has an exponential distribution of run length. A Levy walk is characterized by infinite run length variance, involving a heavy-tailed distribution, often following power law distribution with 1<μ<3. The run length frequency distributions are presented as power plot (Fig. 3E) and exponential plot (Fig. 3F), clearly demonstrating that an exponential plot describes the data much better than a power plot.

In conclusion, the movement of *Dictyostelium* cells is composed of de novo pseudopodia leading to random turns, and of alternating right/left splitting pseudopodia giving rise to the observed relatively straight run of persistent steps. The observed run length probability distribution, turn frequency and autocorrelation strongly suggests that the run length is determined by random selection of split and *de novo* pseudopodia. Consequently, the dispersal of cells is determined by the probability to either extend a splitting pseudopod or a de novo pseudopod.

### Run length during starvation

**Figure 4:**
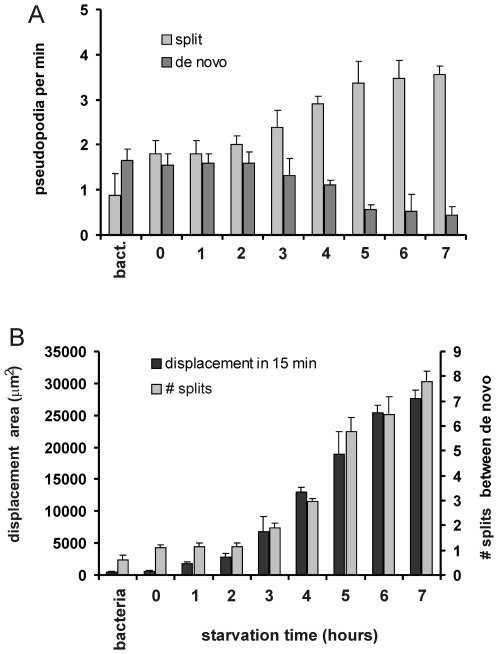
Pseudopod behavior and displacement during starvation. Wild-type AX3 cells were starved for the indicated time. The frequencies of split and *de novo* pseudopodia are presented in panels A. The number of split pseudopodia in between two *de novo* pseudopodia, and the displacement during 15 minutes is presented in panels B. The data show the means and standard deviations of 30 cells from at least two independent movies. See supplemental information Table S1 for additional data.

The repertoire of steps and turns by split and *de novo* pseudopodia may be used to optimize food seeking. As demonstrated in Fig. 2, cells make a transition from intense searching of a small area at 1 h to more open searches of 10-fold larger areas at 5 h of starvation. Fig. 4A reveals that during the first two hours of starvation the split and *de novo* pseudopodia are extended at about equal frequency, leading to ∼1 split in between two *de novo* pseudopodia. After prolonged starvation, the frequency of pseudopod splitting shows a moderate increase, while the frequency of *de novo* pseudopodia decreases strongly, yielding a pronounced increase of the number of split pseudopodia in between two de novo pseudopodia to ∼8 pseudopodia at 7 h of starvation. In conjunction, the dispersal area increases dramatically from 7,000 µm^2^/15 min in 3 hours starved cells to 19,000 µm^2^/15 min in 5 hours starved cells and eventually 28,000 µm^2^/15 min in 7 hours starved cells. Thus, suppression of *de novo* pseudopodia in favor of splitting pseudopodia is used by starved cells as a mechanism to increase the persistence of movement and thereby to disperse over larger areas, which may increase the encounter rate to find new patches of food.

## Discussion

The results show that *Dictyostelium* cells can modulate food searching by changing the probability distribution to extend steps or turns, and then randomly draw from this distribution the steps and turns to make longer or shorter runs. Upon starvation, *Dictyostelium* cells suppress the extension of *de novo* pseudopodia (turns) and enhance the formation of pseudopodia by splitting (steps). We address this strategy as SIRE for Starvation-Induced Run-length Extension. In the past we have identified a cGMP signaling system that is required to suppress *de novo* pseudopodia by forming myosin filaments at the sides and in the back of the cell [16], [19]. The recently discovered PLA2 signaling pathway [20]–[22] also participates in this step/turn modulation [16]. Starved mutant cells lacking the relevant guanylyl cyclase *sGC* and *PLA2A* genes make many turns and few steps; as a consequence, runs are very short and dispersal is extremely poor [16]. The average dispersal of 5 h starved wild-type cells in 15 min is 19,000 µm^2^, which is only 1,600 µm^2^ for 5 h starved *sgc/pla2A*-mutant cells [16]. We propose a simple model for the regulation of the step/turn ratio (Fig. 1): Starvation induces an increase of the activity of guanylyl cyclase and PLA2. cGMP stimulates the formation of myosin filaments in the rear of the cell, thereby locally inhibiting the formation of *de novo* pseudopodia. Pseudopod formation is then more restricted to pseudopod splitting at the front, probably using actin filaments, which is stimulated by PLA2 activity through a mechanism that is not yet known. Split pseudopodia are extended alternating to the right and left at a small angle leading to a relatively straight trajectory.

Recently Li et al. [23] reported on the movement of *Dictyostelium* cells in buffer, with similar but also with very different results from ours. Li et al. determined the position of the centroid of the cell at 10 sec interval during 8 to 10 hours, similar as done by Potel and Mackay [24], while we followed cell displacement at much higher temporal and spatial resolution by detecting the extension of pseudopodia at 1 s intervals during 15 min periods. The three studies agree that movement of starved *Dictyostelium* cells is a persistent random walk with a persistent time of 3 to 8 min. These studies also show that the tail of the probability distribution of turns (Li et al.) or runs (our results) are well explained by an exponential model. However, the change in cell dispersal around 2–3 h of starvation, which is the basis for the SIRE walk, was not investigated by Potel and Mackay, and not mentioned by Li et al., who combined all observations during the entire starvation period from 0 to 10 hours into a single data set. Recently, Takagi et al [25] reported on cell movement of growing and starving *Dictyostelium* cells, demonstrating that starved cells exhibit more persistence than growing cells, in agreement with our observations. However they obtained velocity distributions that had power-law tails, clearly different from all other studies on the movement of *Dictyostelium* cells. Pseudopod extensions were not investigated in any of the previous studies. Therefore, the observed displacements could not be interpreted and quantified in terms of splitting pseudopodia for persistent runs and *de novo* pseudopodia inducing turns.

The function of the SIRE walk as food seeking device was modeled for different amounts of food that is present either homogeneously around the amoebae or in patches at some distance from the cells (see appendix S2 in supplemental information). Cells recover energy from food, which they use for basal metabolism, extension of pseudopodia and movement of the cell body. Cells embedded in a homogeneous food environment may detect the amount of food and adjust their food-seeking device accordingly. When food supplies are ample, a cell can make short runs and small displacements to keep a positive energy balance, but at lower foot densities the model suggests that cells must make long runs resulting in large displacements to collect sufficient food (see Fig. 2A in Appendix S2 of supplemental information). In a heterogeneous environment with food patches, cells do not have information on food availability. Therefore, cells have to invest energy for searches, which they may, or may not, return if finding food. The model shows that cells easily recover the investment when food is close by. However when the patches are a little further away, cells must make long runs otherwise they reach the patches too late for survival (see Fig. 2B in Appendix S2). Although increasing the run length is an efficient way to find food when starved, it is far from optimal, because the best strategy in an infinite environment should be to move in straight lines. So, why do starved *Dictyostelium* cells extend de novo pseudopodia and make occasionally very short runs? Very short runs are the logic consequence of a Bernoulli trial with a finite probability to extend de novo pseudopodia. Cells could prevent very short runs either if they reduce the probability to extend de novo pseudopodia in the Bernoulli trial, or if they do not follow a Bernoulli trial but evolved some memory system by which they reduce the probability to extend a new de novo pseudopod for some period of time after having extended a de novo pseudopod. *Dictyostelium* cells may not have the optimal foraging strategy, because after ∼6 hours of starvation they no longer search for food but develop another strategy to survive, which is cell aggregation by means of chemotaxis to secreted cAMP. The cell aggregate subsequently develops into a fruiting body with spores that can survive without food for very long periods of time. We have analyzed pseudopod extension during chemotaxis (Bosgraaf and Van Haastert, unpublished) and observed that a low frequency of de novo pseudopodia is beneficial for chemotaxis, because cells use de novo pseudopodia to change directions during chemotaxis. Therefore we speculate that efficient chemotaxis forms a trade-off for sub-optimal food searching.

During the sampling process of pseudopodia, we did not find a correlation between split and de novo pseudopodia; the selection of a splitting or de novo pseudopod is independent of the type of pseudopodia in the past. With a characteristic scale of turns, determined by cGMP and PLA2 activity, this results in an exponential probability distribution of run length. We have analyzed several adaptations of this basic mechanism that could improve food searching behavior by SIRE, although we have no strong evidence for their presence in *Dictyostelium*. Incidentally, many of these adaptations lead to runs with less exponential and more power-tail properties (see appendix S1 in supplemental materials). For instance, increasing the probability of a split pseudopod after a longer series of previous splittings would lead to a few exceptionally long runs to new areas where the cell may find food. We did not observe a change in probability distribution of turns up to runs of at least 20 steps (Fig. 3C), but we have not sufficient data to speculate on very long runs. Another strategy for improved food searching is to introduce heterogeneity in a large population of genetically identical cells. We observed that nearly all cells starved for 1–2 hour make many turns that is associated with small displacement. However, in 6 movies with in total 171 cells we observed 5 cells that make very few turns and show large displacement. It is possible that such cells were starved prematurely in growth medium, although we have taken care that cells were harvested at a low cell density when ample food is still available. Alternatively, the large runs of these few cells may be an efficient food seeking strategy of the species, because such pioneering cells move far away from the population of competing cells, and find new patches of food to disperse the genetic content of the *Dictyostelium* clone. A third mechanism of improved SIRE is to correlate the direction of turns, which will increase the persistence and reduces the probability that cells return to earlier visited areas [9]. We observed that de novo pseudopodia (turns) are extended in a direction that is not correlated with the direction of the previous series of splitting pseudopodia or the direction of the previous turn [16]. This suggests that the directional memory of *Dictyostelium* cells does not go beyond the length of one run (turn plus subsequent splittings till the next turn). Thus, instead of correlating the direction of turns to make more persistent trajectories, *Dictyostelium* cells suppress turns by which longer series of persistent splittings are formed. Finally, cells may exhibit composite behavior with periods of long runs and periods of short runs. However, serial autocorrelation of run length of 1 h or 5 h starved cells do not provide any indication for composite behavior (Fig. 3D).

In conclusion, the *Dictyostelium* food searching strategy uses a correlated random walk for movement that is based on persistence by pseudopod splitting and random turns by de novo pseudopodia. We observed only one adaptation to improve food searching, which is to modulate the Bernoulli trial probability distribution of splitting and de novo pseudopodia upon starvation. A relatively simple biochemical-cytoskeleton circuit of starvation-induced cGMP and PLA2 activity suppresses turns and induces splitting, respectively, leading to longer runs. Similar regulatory circuits may be present in other organisms to control the run length during starvation. In addition, such circuits may have been modulated by adaptive control loops to yield more effective food seeking strategies with other statistical properties. A SIRE walk may very well explain the observed differences in search behavior of fed and starving organisms such as bumble-bees [26], flower bug [27], hoverfly [28] and zooplankton [29], that all make longer runs when starved.

## Materials and Methods

### Cells

Wild type AX3 cells were grown in HG5 medium (contains per liter: 14.3 g oxoid peptone, 7.15 g bacto yeast extract, 1.36 g Na_2_HPO_4_⋅12H_2_O, 0.49 g KH_2_PO_4_, 10.0 g glucose), harvested in PB (10 mM KH2PO4/Na2HPO4, pH 6.5), and allowed to develop in 1 ml PB in a well of a 6-wells plate (Nunc). Movies were recorded with an inverted light microscope (Olympus Type CK40 with a LWD A240 20x numerical aperture 0.4 objective) fitted with a JVC TK-C1381 CCD camera. Digital images were captured at a rate of 1 frame/s on a PC using VirtualDub software and Indeo video 5.10 compression. Cells can freely move in an area of 7 cm^2^; the field of observation was 358×269 µm.

### Data analysis

Images were analyzed using an automated computer algorithm Quimp3. The program identifies pseudopodia, defines the position and time of the tip of the pseudopod at the beginning and end of its growing phase, and annotates pseudopodia as maintained versus retracted, and split versus de novo [15]. The field of observation contains about 20 cells. Cells that do not stay in the field during the 15 minutes of the movie were ignored. Of the remaining cells we determined the displacement. For pseudopod analysis we ignored the cells with poor phase contrast and cells that move very slow or extremely fast. Of the remaining ∼12 cells we selected 3–5 cells that are easily analyzed by the computer algorithm (minimal contact with other cells and good phase contrast), and have a dispersion close to the average dispersion of the population. Cells extend 3–4 pseudopodia per minute, providing data on 40–60 pseudopodia per cell. The array of *de novo* and split pseudopodia of one cell contains ‘closed’ runs (a series of split pseudopodia in between two *de novo* pseudopodia), but the array begins and ends with ‘open’ runs (no de novo pseudopodia at the beginning and at the end of the array). To use these open runs, the pseudopod data of all cells from a specific developmental stage were placed in a circular array so that the open end of one cell is closed by the open start of the next cell. A typical database contains information on 200–300 pseudopodia obtained from 6–10 cells from two independent movies. For Fig. 3 we collected additional data for vegetative (426 pseudopodia) and 5 h starved cells (1645 pseudopodia). The data are presented as the means and standard error of the means (SEM) where n represents the number of cells analyzed, or as the means and standard deviation (SD) where n represents the number of pseudopodia analyzed.

### Analysis for random turns

As explained in the introduction, the movement of *Dictyostelium* cells can be described by relatively straight runs of pseudopod splittings and turns by de novo pseudopodia. A run with length (*r*) consists of one de novo pseudopod and the subsequent (*r*-1) splitting pseudopodia until the next de novo pseudopod. We have analyzed whether turns occurs randomly, or whether the probability of turns may increase or decrease when cells have extended longer series of splitting pseudopodia. Therefore, the probability of a turn was calculated at increasing length of the run according to:
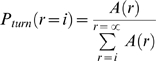
(1)where *A(r)* is the number of runs with length *r* pseudopodia.

In addition, we calculated the serial autocorrelation of *Δr* to find possible clusters of shorter or longer run length that may occur in composite random walks. Here *Δr* is the fractional deviation the run length *r(n)* from the mean the run length; *Δr*  =  (*r(n)*−<*r*>)/<*r*>. For a series of N runs with run numbers n, the autocorrelation of *Δr* was calculated according to:
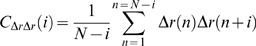
(2)where 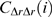
indicates the correlation between the deviation from mean run length *Δr*(*n*) at run number *n* with the deviation from mean run length *Δr*(*n* + i) at run number n+i. Short runs have negative *Δr*, whereas long runs have positive *Δr*; therefore clusters of short runs (as well as clusters of long runs) have a positive 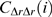
for a small interval *i*.

## Supporting Information

Table S1 Pseudopod properties of Dictyostelium cells during development.
(0.05 MB PDF)

Appendix S1 Equations describing movement of cells with random turns and persistent steps
(0.16 MB PDF)

Appendix S2 Growth and death at different run length
(0.19 MB PDF)
