# Supplementary material for: Food Searching Strategy of Amoeboid Cells by Starvation Induced Run Length Extension
Source: PLoS One. 2009 Aug 28;4(8):e6814. doi: 10.1371/journal.pone.0006814 (PMC2729374; doi:10.1371/journal.pone.0006814)
Supplement: Table S1 — Pseudopod properties of Dictyostelium cells during development. (0.05 MB PDF) [file pone.0006814.s001.pdf]

Table S1 Pseudopod properties of *Dictyostelium* cells during development

Strain	n	Pseudopod size (µm)		Pseudopod growth time (s)		Pseudopod growth speed (µm/min)		Pseudopod interval (s)		Cell speed (µm/min)		Pseudopod activity (µm/min)		Pseudop retraction (%)	Forward pseudop activity (µm/min)	Forward pseudop activity/ speed	Frequency Split Pseudopodia (1/s)		Frequency *de novo* pseudopodia (1/s)		Persistence (a, # split pseudopodia)	
		mean	SEM	mean	SEM	mean	SEM	mean	SEM	mean	SEM	Mean	SEM	mean	mean	mean	mean	SEM	mean	SEM	mean	SEM
WT bacteria	7/191	3.76	0.08	13.10	1.41	0.29	0.01	21.96	3.20	2.07	0.24	10.28	0.86	58.33	4.28	2.07	0.87	0.48	1.65	0.26	0.63	0.20
WT 0h	8/231	3.97	0.17	10.46	1.02	0.38	0.03	20.15	2.81	5.46	1.02	13.36	2.47	24.75	8.90	1.63	1.79	0.30	1.55	0.24	1.15	0.10
WT 1h	7/215	5.03	0.21	12.12	0.76	0.41	0.03	20.00	1.43	8.85	0.46	15.09	1.21	17.19	12.50	1.41	1.80	0.30	1.60	0.20	1.20	0.15
WT 3h	8/256	5.30	0.20	13.21	0.21	0.36	0.01	21.31	0.85	7.39	0.65	15.01	0.52	15.63	12.59	1.70	2.38	0.38	1.31	0.38	1.98	0.17
WT 5h	12/323	5.24	0.17	12.95	0.31	0.49	0.02	16.01	0.17	8.53	0.64	19.63	2.05	13.61	16.97	1.99	3.37	0.48	0.56	0.10	6.00	0.58
WT 7h	7/294	4.67	0.23	10.15	0.53	0.50	0.02	15.49	1.07	9.12	0.70	18.09	1.70	14.50	15.47	1.70	3.56	0.19	0.44	0.19	8.07	0.43

n is the number of experiments; two values are given, the number of cells and the number of pseudopodia, respectively. Data were obtained from two movies for each developmental time point. Cell speed is the instantaneous speed measured at 8s per frame. Pseudopod activity is the total size of pseudopodia extended per minute, and given by 60* (pseudopod size)/ (pseudopod interval). The % retraction indicates the fraction of pseudopodia that are retracted before the pseudopod is split. The forward pseudopod activity is the activity of the pseudopodia that contribute to movement of the cell and is given by (pseudopod activity)* (100-% retraction)/100.

Movement of cells in bacteria is very slow. The reason could be few pseudopodia (not observed), small pseudopodia (not observed) or most pseudopodia are retracted (observed). The retracted pseudopodia do not contribute to movement of the cell.
