# Supplementary material for: Food Searching Strategy of Amoeboid Cells by Starvation Induced Run Length Extension
Source: PLoS One. 2009 Aug 28;4(8):e6814. doi: 10.1371/journal.pone.0006814 (PMC2729374; doi:10.1371/journal.pone.0006814)
Supplement: Appendix S1 — Equations describing movement of cells with random turns and persistent steps (0.16 MB PDF) [file pone.0006814.s002.pdf]

Appendix S1. Equations describing movement of cells with random turns and persistent steps

A *Dictyostelium* cell can move with two types of pseudopodia, by splitting of an existing pseudopod or by extending of a *de novo* pseudopod, respectively [1]. Pseudopod splitting is predominantly alternating right/left steps at ~60 degrees by which cells move in a nearly straight zig-zag fashion. *De novo* pseudopodia are extended in nearly random directions, thereby interrupting the straight path of splitting pseudopodia [2]. Splitting pseudopodia are addressed as *s* (for steps), while *de novo* pseudopodia providing random directions are addressed as *t* (for turn). The more or less straight path with splitting pseudopodia in between two turns is called a run and consists of *r* pseudopodia.

A run of *r* pseudopodia starts with a turn (giving the first pseudopod), followed by r-1 steps, and terminated by a turn. The frequency of *s* is σ, and the frequency of *t* is 1-σ. Note that σ depends on the mean run length $<r>$ as $\sigma = (< r > -1) / < r >$. The probability *P(r)* to observe a run with *r* pseudopodia is given by

$$P(r) = p = (1-\sigma)^2 \sigma^{r-1} \qquad \text{(Eq. S1)}$$

In a long series of *n* pseudopodia the probability to observe *k* runs of length r is binomial distributed. Since we are interested in long runs, which have small probabilities, the Poisson distribution is a good approximation of the binomial distribution. The probability *P(r,k)* to observe *k* runs with *r* length in a series of *n* pseudopodia is

$$P(r,k) = \frac{e^{-np}(np)^k}{k!} \qquad \text{(Eq. S2)}$$

The number of observed runs *A(r,k)* with length *r* and with *k* successes is

$$A(r,k) = k\frac{e^{-np}(np)^k}{k!} \qquad \text{(Eq. S3)}$$

and the total number of observed runs *A(r)* with length *r* is

$$A(r) = \sum_{k=1}^{k=n-r} k\frac{e^{-np}(np)^k}{k!} \qquad \text{(Eq. S4)}$$

The frequency of observed runs with length *r* is then given by

$$F(r) = \frac{A(r)}{\sum_{r=1}^{r=n} A(r)} \qquad \text{(Eq. S5)}$$

Special cases of the model which tend to power tail distributions.

In *Dictyostelium* the starvation-induced extension of the run length is relatively modest and slow. The average run length increases with approximately one pseudopod per hour. We have analyzed steps and turns during only 15 minutes, and therefore the obtained data are probably an accurate description of the probability frequency distributions of steps and turns, and consequently of the run length. The results show that the probability of turns is time-independent, and therefore results in an exponential distribution (figure 1), as has been observed experimentally (Fig. 3E,F of main manuscript). However, there may be situations in which the data set are less robust. Here we analyzed two effects: First, the situation where the data are not collected during the short interval of 15 min, but during the entire 8 hours period of starvation [3], and second, the situation where the population of cells may not be homogeneous [4].

Measurement during 8 hours. Cells extend about 180 pseudopodia per hour and runs longer than 10 minutes (30 pseudopodia) are rare ($<0.2\%$). Therefore, the determination of the run length frequency during 15 minutes occurs at nearly constant probabilities of split and turn pseudopodia. However, when the run length would be measured during the entire period of 8 hours starvation with increasing mean run length from $<r> = 2$ to $<r> = 9$, the obtained probability frequency distribution will be complex. Short runs originate predominantly during early starvation period and long runs during late starvation. As a consequence, the probability frequency distribution is no longer true exponential, and has characteristics intermittent between a power function and exponential function (see the blue line in figure 1).

Non-homogeneous cell population. Cells starved for one hour move with low persistence because they extend not many splitting pseudopodia. We noticed that movies of early starved cells contained a few cells that moved with strong persistence. These cells exhibited many splitting pseudopodia and had a large displacement, as if they were starved for a much longer period. These cells were not included in our analysis, because they have a displacement much larger than the average displacement of the population (see methods). We calculated the effect of a heterogeneous cell population on the frequency distribution of run length for a mixture of 90% cells with $<r> = 2$ (1 hour starved) and 10% cells with $<r> = 7$ (5 hours starved). The results (see the red line in figure 1) show that also in this situation the probability frequency distribution is no longer true exponential, and has characteristics intermittent between a power function and exponential function.

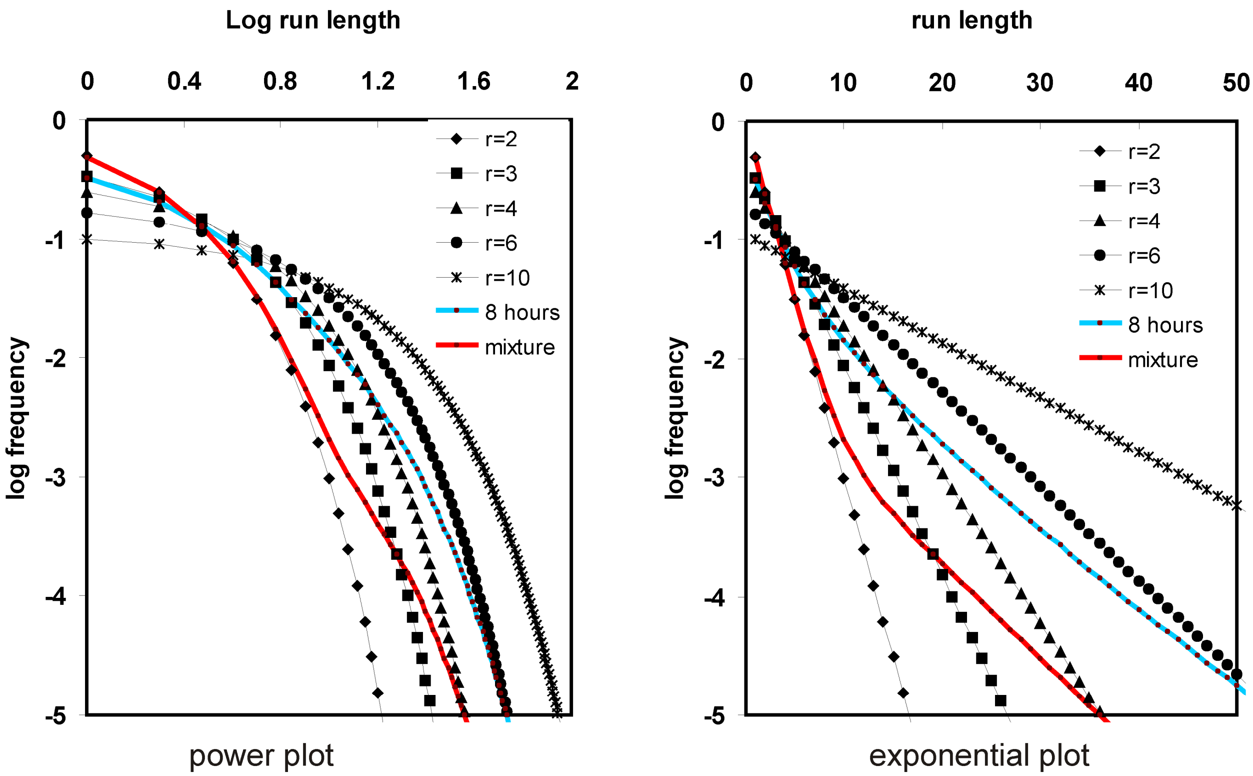

Figure 1. Model calculations of the probability frequency distribution of runs length. The curves were calculated for different mean run length using with equations S1-S5. Data are plotted as power plot (left) and as exponential plot (right). The blue curve is the probability distribution for 8 phases with increasing mean run length from <*r*> = 2 to <*r*> = 9. The red line represents the probability distribution of 90% cells with <*r*> = 2 and 10% of cells with <*r*> = 6.

References

1. Andrew, N., and Insall, R.H. (2007). Chemotaxis in shallow gradients is mediated independently of PtdIns 3-kinase by biased choices between random protrusions. Nat Cell Biol *9*, 193-200.
2. Bosgraaf, L., and van Haastert, P.J.M. (2009). The Ordered Extension of Pseudopodia by Amoeboid Cell in the Absence of external Cues. PLoS ONE *4*, e5253.
3. Li, L., Norrelykke, S.F., and Cox, E.C. (2008). Persistent cell motion in the absence of external signals: a search strategy for eukaryotic cells. PLoS ONE *3*, e2093.
4. Takagi, H., Sato, M.J., Yanagida, T., and Ueda, M. (2008). Functional analysis of spontaneous cell movement under different physiological conditions. PLoS ONE *3*, e2648.
