# Supplementary material for: Food Searching Strategy of Amoeboid Cells by Starvation Induced Run Length Extension
Source: PLoS One. 2009 Aug 28;4(8):e6814. doi: 10.1371/journal.pone.0006814 (PMC2729374; doi:10.1371/journal.pone.0006814)
Supplement: Appendix S2 — Growth and death at different run length (0.19 MB PDF) [file pone.0006814.s003.pdf]

Appendix S2. Growth and death at different run length

When a cell moves it will occupy a certain area where it may find food. The food is used for basal cell metabolism and for movement. When more food/energy is found than needed for metabolism and movement the cell will grow, while the cell will die when it used more energy that taken up from the food. We will recognize two situations, when the food is homogeneously distributed around the cell, and when the food is present heterogeneously in small patches, respectively. In both cases dispersion of the cell depends on the diffusion rate constant D and time t, while energy expenditure per unit of time depends on basal metabolism (α), extension of pseudopodia (β) and actual displacement of the cell (γ).

A. Diffusion rate constant during starvation.

Movement of *Dictyostelium* cells at short time intervals is a persistent random walk, which converts to a random walk after 1-5 minutes. Since we estimate here growth and survival after several hours, movement in two dimensions is accurately described by a random walk with

$$\langle L^2 \rangle = 4Dt \qquad \text{(Eq. S6)}$$

where $\langle L^2 \rangle$ is the mean square displacement, and D is the diffusion rate constant.

We determined the diffusion rate constant D and the mean run length <*r*> for different starvation times (see table 2), showing that $D = 2.55 < r >^2$.

Table 2. Mean run length and diffusion rate constant of starving *Dictyostelium* cells

Starvation time (hours)	$<r>$ (# of pseudopodia)	D (μm^2/min)
0	2.1	3.19
1	2.2	9.76
2	2.2	15.78
3	3.0	38.76
4	4.9	77.57
5	7.0	119.16
6	7.7	162.15
7	8.7	181.40

B. Food searching in homogeneous environment

We assume that *Dictyostelium* cells are in a homogeneous environment where they diffuse (thereby visit new area with food), and takes up food proportional to the new area visited and food density ρ. The food taken up is given by:

$$dF / dt = b < r >^2 \rho \qquad \text{(Eq. S7)}$$

Where b is a constant ($b = 4\pi 2.55$) and <*r*> is the mean run length.

The food is used for basal metabolism (α), pseudopod extension (β for all pseudopodia) and displacement of the cell (γ for split pseudopodia).

$$dS / dt = \alpha + \beta\theta + \gamma\theta \frac{< r >}{< r > +1} \qquad \text{(Eq. S8)}$$

where θ is the pseudopod frequency. The energy balance is then given by

$$dE / dt = dF / dt - dS / dt = b < r >^2 \rho - \alpha - \beta\theta - \gamma\theta \frac{< r >}{< r > +1} \qquad \text{(Eq. S9)}$$

We calculated the amount of food that is needed to recover the energy spend during the flight for different values for the mean run length <*r*>. Figure 2A reveals that cells in a homogeneous environment can make short runs and small displacements at high food density, and still keep a positive energy balance. At a lower foot densities the model suggests that cells must make long runs resulting in large displacements to collect sufficient food.

C. Food searching in heterogeneous environment

We assume that *Dictyostelium* cells are in an area devoid of food except in a small area at a distance *l* from the cell. The cell will diffuse and when it reached the food spot will become adsorbed. The cell will have to invest energy for movement, and may recover energy when the food spot is found.

The probably density function (PDF) of the time it takes for a cell that diffuses in two dimensions to become adsorbed at a distance *l* (see [1])

$$PDF(t) = \frac{l^2}{4Dt^2} e^{-l^2 / 4Dt} \qquad \text{(Eq. S10)}$$

The uptake of food at time *t* is then the probability that the cell has been adsorbed multiplied by the amount of food ρ, while the energy spend during time period *t* is given by equation S8. Therefore, the energy balance at time *t* is given by

$$E(t) = \rho \frac{l^2}{4Dt^2} e^{-l^2 / 4Dt} - (\alpha + \beta\theta + \gamma\theta \frac{< r >}{< r > +1})t \qquad \text{(Eq. S11)}$$

We calculated the amount of food at the target spot that is needed to recover the energy spend during the flight for different values for the target distance *l* and the mean run length <*r*>. The model shows that cells easily recover the investment when food is close by. However when the patches are a little further away, cells must make long runs otherwise they reach the patches too late for survival (Fig 2B).

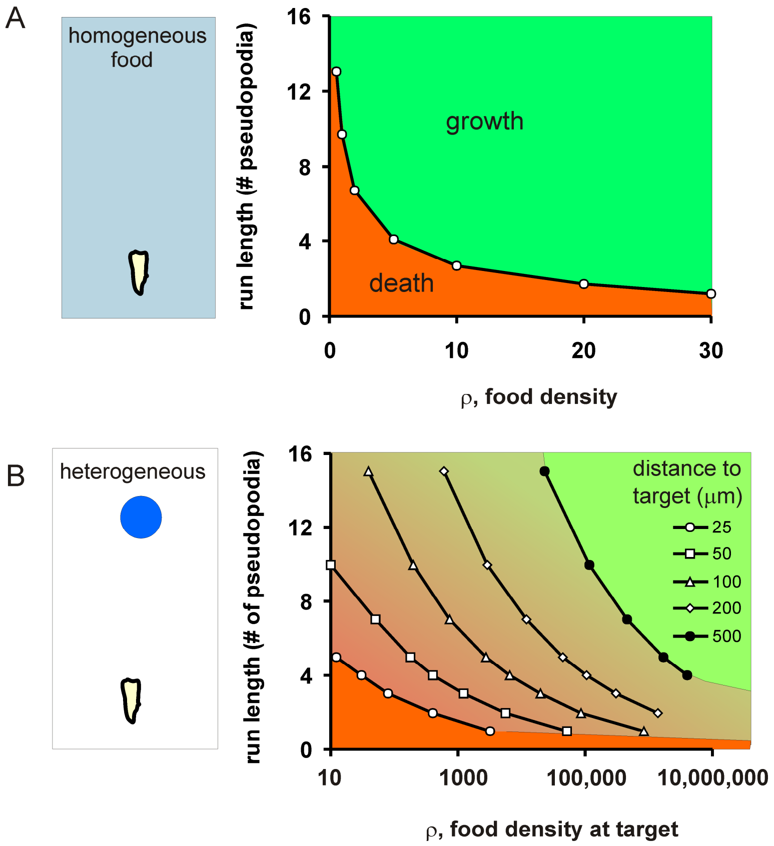

Figure 2. Death and growth by SIRE, Starvation-Induced Run-length Extension. Model calculations on the effect of run length on the energy balance of cells in an environment with homogeneous food (**A**) and food in patches (**B**) using equations S9 and S11, respectively. Parameter selection: cells take up bacteria that are present at density ρ (in arbitrary energy quanta per μm^2), and use energy for basal metabolism (α = 100 min^{-1}), pseudopod extension (β = 200 min^{-1}) and displacement of the cell (γ = 400 min^{-1}). The surface area of the patch is 1000 μm^2.

REFERENCES

1. Codling, E.A., Plank, M.J., and Benhamou, S. (2008). Random walk models in biology. J R Soc Interface *15*, 15.
